# Effects of Microplastics on Growth, Photosynthesis, and Oxidative Stress-Related Traits in Terrestrial Plants: A Global Meta-Analysis

**DOI:** 10.3390/plants15192911

**Published:** 2026-09-23

**Authors:** Jinling Xu, Dongwen Yu, Mufan Liu, Xuehan Zhang, Hangyu Liu, Yuzhen Liu

**Affiliations:** 1 College of Geography and Environment, Shandong Province Key Laboratory of Emerging Contaminants Risk Prevention and Control, Shandong Normal University, Jinan 250358, China; 18853596909@163.com (D.Y.); 2025215014@stu.sdnu.edu.cn (X.Z.); 110102@sdnu.edu.cn (Y.L.); 2 College of Life Sciences, Shandong Normal University, Jinan 250358, China; 202513000237@stu.sdnu.edu.cn; 3 College of Natural Resources and Environment, Northwest A&F University, Yangling 712100, China; 06091836@nwafu.edu.cn

**Keywords:** microplastics, plant ecophysiology, plant functional groups, biodegradable microplastics, soil properties, random forest

## Abstract

Microplastics (MPs) can affect growth, photosynthetic, and oxidative stress-related traits in plants, but the reported effects vary among studies. However, the roles of plant groups, MP characteristics, soil properties, and exposure conditions have not been systematically compared across these three trait groups. We compiled 2613 paired observations from 100 independent studies and combined meta-analysis with random forest modeling to quantify MP effects on the three trait groups and compare the relative importance of candidate predictors. Overall, MPs increased oxidative stress-related traits by 19.92% and reduced photosynthetic and growth traits by 12.87% and 12.31%, respectively. Plant-group differences were trait-specific, with numerically larger root reductions in monocotyledons, a larger numerical decline in shoot fresh biomass in dicotyledons, and stronger POD and SOD responses in monocotyledons. Higher MP concentrations corresponded to larger reductions in several biomass traits and plant height. The effects of particle size were less consistent. Biodegradability showed limited differences across traits, with a significant difference detected only for H_2_O_2_. Temperature was related to trait responses across all three groups and also ranked highly in the random forest analysis. These results improve our understanding of differences in plant responses to MPs and provide a basis for evaluating MP effects across plant groups, MP characteristics, soil properties, and exposure conditions.

## 1. Introduction

Plastic pollution is widespread in water, air, and terrestrial environments and is difficult to reverse once released [1]. Microplastics (MPs) are commonly defined as plastic particles smaller than 5 mm [2]. Early research focused mainly on marine and freshwater systems, whereas plastic pollution in soils received much less attention [3]. Agricultural soils receive MPs from mulch residues, plastic-containing soil amendments, irrigation water, and atmospheric deposition [4]. MPs in soil can alter water movement, soil aggregation, and microbial activity and may consequently affect plant growth [5,6].

Small plastic particles taken up by plants may subsequently enter terrestrial food webs. In wheat and lettuce, small plastic particles entered roots through cracks at sites of lateral-root emergence and moved to the shoots under transpirational pull [7]. A tracing study using 200 nm polystyrene (PS) particles found that most particles remained in the roots and only a small proportion reached the shoots [8]. Leaves provide another entry route, as airborne MPs can be absorbed and accumulate in plant tissues [9]. Experimental studies have recorded changes in plant biomass, photosynthetic pigments and gas exchange, oxidative damage markers, and antioxidant enzyme activities under MP exposure [10,11].

Recent meta-analyses have shown that plant responses to MPs vary with polymer type, particle size, concentration, and plant species, but conclusions regarding the effects of MP concentration and particle size remain inconsistent [11,12,13,14]. For example, Shi et al. [11] observed greater reductions in root activity and biomass at higher MP concentrations and under exposure to smaller particles. By contrast, Huo et al. [14] found less consistent concentration–response relationships in soil-grown plants, with both positive and negative changes in growth, germination, and chlorophyll content. Wang et al. [12] showed stronger inhibition of photosynthetic rate but a smaller reduction in chlorophyll *a* with increasing particle size, while hydrogen peroxide (H_2_O_2_) and antioxidant enzyme activities also differed among particle-size classes. The effects of MPs on plants also differ among plant species and polymer types. Zantis et al. [15] directly compared weathered polyethylene (PE) and a starch-based poly(butylene adipate-co-terephthalate) (PBAT) blend in two monocots, barley and wheat, and two dicots, carrot and lettuce. Adverse effects during early development were more common in carrot and lettuce than in barley and wheat and under PBAT exposure than under PE exposure. However, systematic comparisons of MP effects between plant groups and between biodegradable and non-biodegradable MPs remain limited across growth, photosynthetic, and oxidative stress-related traits.

MP effects on plants can also vary with soil properties and exposure conditions. The same 1% treatment with large polyurethane (PU) MPs increased maize dry biomass in lime concretion black soil but reduced it in silty loam soil [16]. Guo et al. [17] found that nutrient-rich conditions promoted root nutrient foraging and masked some negative effects of MPs, whereas under nutrient-poor conditions, MP-induced soil dehydration reduced photosynthetic performance and increased competition for limited nutrients. Temperature can affect photosynthetic carbon (C) assimilation, reactive oxygen species (ROS) production, and antioxidant defense [18]. Wang et al. [12] also noted that differences in light intensity may partly explain the different chlorophyll responses reported among studies. However, how plant responses to MPs vary with soil properties and exposure conditions remains less clear, and the relative importance of these factors has rarely been compared across trait groups.

In this study, we compiled 2613 paired observations from 100 independent studies to (1) quantify the effects of MPs on plant growth, photosynthetic traits, and oxidative stress-related traits; (2) examine how these effects varied with plant groups, MP characteristics, soil properties, and exposure conditions; and (3) compare the relative importance of candidate predictors in explaining variation in MP effect sizes across the three trait groups using random forest models.

## 2. Results

### 2.1. Overall Effects of MPs on Growth, Photosynthetic, and Oxidative Stress-Related Traits

Plant growth and photosynthetic traits showed overall decreases under MP exposure, whereas oxidative stress-related traits showed an overall increase (Figure 1). Specifically, growth and photosynthetic traits decreased by 12.31% (95% CI: −16.40% to −8.03%) (Figure 1a) and 12.87% (95% CI: −20.43% to −4.60%) (Figure 1b), respectively, whereas oxidative stress-related traits increased by 19.92% (95% CI: 8.27% to 32.83%) (Figure 1c). Among the growth traits, shoot fresh biomass showed the largest decrease (−20.70%), followed by root fresh biomass (−15.13%), shoot dry biomass (−14.13%), root dry biomass (−13.77%), and plant height (−9.65%). Root length decreased by 6.25%, but the change was not significant (Figure 1a). For photosynthetic traits, net photosynthetic rate, stomatal conductance, transpiration rate, chlorophyll *b*, chlorophyll *a*, and intercellular CO_2_ concentration decreased by 30.45%, 25.70%, 13.96%, 13.86%, 8.22%, and 5.60%, respectively. The decreases in transpiration rate, chlorophyll *a*, and intercellular CO_2_ concentration were not significant (Figure 1b). Under MP exposure, H_2_O_2_ and malondialdehyde (MDA) contents increased by 43.80% and 30.30%, respectively. The activities of catalase (CAT), peroxidase (POD), and superoxide dismutase (SOD) increased by 25.68%, 19.91%, and 11.97%, respectively, although the changes in H_2_O_2_, CAT, and SOD were not significant.

### 2.2. Responses of Monocotyledons and Dicotyledons to MPs

Responses to MPs differed between dicotyledons and monocotyledons for some traits (Figure 2, Table S4). For growth traits, monocotyledons showed larger numerical reductions in root dry biomass (−15.90%) and root length (−13.56%) than dicotyledons (−12.53% and −2.24%, respectively) (Figure 2a). By contrast, shoot fresh biomass decreased more in dicotyledons (−22.57%) than in monocotyledons (−13.98%). Shoot dry biomass and plant height showed similar reductions in dicotyledons and monocotyledons (−14.19% vs. −14.08% and −9.44% vs. −9.72%, respectively). None of the growth traits showed a significant difference between the two plant groups. For photosynthetic traits, monocotyledons showed larger decreases in chlorophyll *a* (−15.70% vs. −6.96%) and stomatal conductance (−34.66% vs. −20.20%) than dicotyledons, although these differences were not significant. Intercellular CO_2_ concentration decreased by 21.60% in monocotyledons but changed little in dicotyledons (+1.10%), and this difference was significant (*p* = 0.038) (Figure 2b). Oxidative stress-related traits generally showed larger increases in monocotyledons than in dicotyledons. The differences were significant for POD (54.34% vs. 7.92%, *p* = 0.034) and SOD (39.40% vs. 3.79%, *p* = 0.049), whereas H_2_O_2_, MDA, and CAT did not differ significantly between the two plant groups (Figure 2c).

### 2.3. Effects of MP Biodegradability, Concentration, and Particle Size

Biodegradable MPs showed larger numerical reductions in some growth traits than non-biodegradable MPs (Figure 3). Root dry biomass and shoot dry biomass declined by 21.03% and 19.24% under biodegradable MPs, compared with 12.31% and 13.39% under non-biodegradable MPs, although the differences between MP types were not significant. Among the oxidative stress-related traits, H_2_O_2_ increased more under non-biodegradable MPs than under biodegradable MPs (51.92% vs. 20.90%, *p* = 0.044).

Root dry biomass decreased by 8.34%, 7.31%, and 17.80% at <0.1%, 0.1–1%, and ≥1%, respectively (*p* = 0.018). Differences among concentration classes were also observed for shoot dry biomass (−9.98%, −6.31%, and −20.67%; *p* = 0.007), shoot fresh biomass (−12.94%, −14.67%, and −32.10%; *p* < 0.001), and plant height (−6.59%, −7.23%, and −13.75%; *p* = 0.009). Among the photosynthetic traits, chlorophyll *b*, net photosynthetic rate, and stomatal conductance also differed among concentration classes (*p* < 0.05). Among the oxidative stress-related traits, SOD activity differed significantly among concentration classes and showed the largest increase at ≥1% (23.37%; *p* = 0.006). MDA increased from 20.21% at <0.1% to 39.92% at ≥1%, although the differences among concentration classes were not significant (*p* = 0.215).

Particle size did not show a consistent relationship with most traits. Root length decreased by 12.64% at 0–10 μm and 13.08% at ≥100 μm, but increased by 11.22% at 10–100 μm (*p* < 0.001). No significant differences among particle-size classes were observed for the other growth and oxidative stress-related traits with sufficient replication. Stomatal conductance also differed among particle-size classes (*p* = 0.021).

### 2.4. Effects of Soil Properties on Plant Responses to MPs

The effects of soil pH and soil organic matter (SOM) on growth and oxidative stress-related traits under MP exposure are shown in Figure 4. Most growth traits did not differ significantly among soil pH classes. Among the oxidative stress-related traits, CAT was the main exception, increasing by 88.38% at pH 6.5–7.5 and by 27.50% at pH ≥ 7.5, but decreasing by 19.64% at pH < 6.5 (*p* = 0.027) (Figure 4b). SOM was related to the responses of some growth traits. Shoot dry biomass decreased by 28.90% at <15 g kg^−1^ and by 18.14% at ≥25 g kg^−1^, whereas little change was observed at 15–25 g kg^−1^ (+1.80%; *p* = 0.004). Plant height showed a similar response, decreasing by 13.44% and 7.31% at low and high SOM levels, respectively, but increasing slightly at 15–25 g kg^−1^ (+4.01%; *p* = 0.040). No significant differences among SOM classes were detected for the other growth and oxidative stress-related traits.

### 2.5. Effects of Exposure Conditions on Plant Responses to MPs

The effects of exposure conditions on plant responses to MPs are shown in Figure 5. Most traits did not differ significantly between the two photoperiod groups. Shoot dry biomass decreased by 21.93% at 13–24 h and by 5.57% at <13 h (*p* < 0.05). Root dry biomass also decreased more at 13–24 h, although the difference between photoperiod groups was not significant (*p* = 0.069).

Several growth, photosynthetic, and oxidative stress-related traits differed significantly among temperature classes. Shoot dry biomass decreased by 26.39% at ≥25 °C compared with 5.27% at 20–25 °C (*p* = 0.002). Larger changes in root biomass, chlorophyll contents, H_2_O_2_, MDA, and antioxidant enzyme activities were observed at <20 °C, although some of these estimates were based on only one independent study.

Most traits did not differ significantly between illumination classes. Transpiration rate differed between illumination classes, although only one to two independent studies were available for each class. No significant differences were detected between the two exposure-duration groups. Root dry biomass decreased by 19.15% at ≥45 d and by 8.43% at <45 d, while SOD increased more during the shorter exposure period, but neither difference was significant.

### 2.6. Relative Importance of Predictors for MP Effects on Plant Traits

Random forest analysis showed that the leading predictors differed among trait groups (Figure 6). Temperature had the highest mean normalized importance for growth and oxidative stress-related traits, with values of 26.58% and 32.75%, respectively, whereas SOM ranked highest for photosynthetic traits (31.56%). MP size and concentration also ranked highly for growth, while SOM, soil pH, and MP concentration were more prominent for oxidative stress-related traits. Ranking stability across the outer cross-validation folds was assessed using mean rank, rank interquartile range, and Top-3 and Top-5 frequencies (Table S9). Temperature remained relatively stable for growth and oxidative stress-related traits, whereas SOM was the most stable predictor for photosynthetic traits. Cross-validated predictive performance was limited (*R*^2^ = −0.043, −0.234, and −0.173 for growth, photosynthetic, and oxidative stress-related traits, respectively; Table S8). The random forest analysis was therefore used mainly to compare the relative importance of the candidate predictors.

## 3. Discussion

### 3.1. Differential Sensitivity of Plant Traits to MP Exposure

Our meta-analysis showed that oxidative stress-related traits changed more strongly than photosynthetic and growth traits under MP exposure. MDA content and POD activity increased significantly, whereas H_2_O_2_, CAT, and SOD also increased numerically but the pooled changes were not significant. These responses indicate that oxidative injury and antioxidant defense occurred together under MP exposure. Jia et al. [19] also reported ROS accumulation and changes in antioxidant activity under MP stress, with the responses varying among plant species and tissues and with MP type, concentration, and exposure duration. When ROS production exceeds removal, ROS can accumulate and damage cellular lipids, proteins, and DNA [20]. The increase in antioxidant enzyme activities observed here therefore reflects activation of the plant antioxidant system in response to MP stress.

Within the photosynthetic group, net photosynthetic rate and stomatal conductance showed the largest reductions under MP exposure. Intercellular CO_2_ concentration changed relatively little and its decrease was not significant, suggesting that stomatal limitation alone may not fully explain the decline in photosynthesis. Mesophyll conductance and metabolic capacity can also constrain photosynthesis under stress [21]. Chlorophyll b decreased significantly, whereas chlorophyll a showed a smaller decrease. Jia et al. [19] reported that MPs can reduce chlorophyll contents and leaf gas exchange and disturb redox reactions and electron transfer in photosystem II (PSII), providing additional pathways for photosynthetic inhibition.

The overall reductions in growth and photosynthetic traits were similar, although individual growth traits responded differently. Shoot fresh biomass showed the largest reduction, whereas root length did not change significantly. Biomass accumulation depends on whole-plant C gain and its allocation among organs, and respiration, cellular maintenance, and stress defense also consume part of the energy acquired through photosynthesis [22,23]. This helps explain why changes in photosynthesis were not reflected equally across all growth traits.

### 3.2. Differences Between Monocotyledons and Dicotyledons Under MP Exposure

Monocotyledons and dicotyledons did not differ consistently in growth responses to MP exposure. Monocotyledons tended to show stronger root inhibition, while clearer between-group differences occurred for some physiological traits, particularly intercellular CO_2_ concentration and the antioxidant enzymes POD and SOD. Many monocot crops, especially cereals, develop fibrous root systems and produce additional axial roots from the stem, whereas dicot crops generally retain a primary root with several orders of lateral roots [24,25]. These differences may affect how roots respond to changes in the surrounding soil. Cereal roots lack a vascular cambium and therefore rely more on continued root growth to increase xylem and phloem tissues, whereas dicot roots can increase transport capacity through secondary thickening [24]. Bramley et al. [26] showed that wheat had a much longer root system than lupin but lower hydraulic conductance, indicating that greater root length does not necessarily provide greater water transport capacity. This distinction may be relevant under MP exposure because MPs can alter soil water availability and other physical conditions around roots. In maize, MP-induced reductions in soil water availability were associated with restricted root water and nutrient uptake and reduced photosynthetic performance [17].

Particle-tracing studies do not show consistently greater uptake or root-to-shoot transport in monocotyledons. Cucumber accumulated more particles in exposed roots than maize, whereas wheat showed lower root-to-shoot translocation than carrot and lettuce [27,28]. Particle entry also depends on local root features. Small particles can enter through cracks formed during lateral-root emergence [7], and root wounds can provide additional entry routes [29]. Greater MP uptake alone is therefore unlikely to explain the stronger physiological responses observed in monocotyledons. MP exposure can also alter root anatomy; increased aerenchyma formation has been reported in maize [16]. The larger increases in POD and SOD instead point to differences in oxidative-stress responses between the two plant groups.

### 3.3. Effects of MP Characteristics on Plant Responses

Biodegradability did not produce a consistent difference in plant responses. Most growth and photosynthetic traits were similar between biodegradable and non-biodegradable MPs, although root dry biomass showed a larger numerical decline under biodegradable MPs. H_2_O_2_ was the only oxidative trait that differed significantly between the two groups, with a larger increase under non-biodegradable MPs. Individual experiments have nevertheless reported stronger effects of biodegradable MPs in some soil–plant systems [30,31,32]. In soybean and wheatgrass, biodegradable MPs increased labile or dissolved organic C and altered soil N availability, accompanied by poorer plant growth [30,32]. Similar effects were reported in lettuce, where PBAT MPs had stronger effects on oxidative damage, photosynthesis, soil aggregation, microbial activity, and ammonium availability than PE MPs [31]. These soil responses may help explain some of the plant effects reported in individual experiments, but they were not tested directly in our meta-analysis.

Root dry biomass, shoot dry biomass, shoot fresh biomass, and plant height showed the largest reductions at the highest concentration, whereas photosynthetic and oxidative responses were less uniform. Experimental studies have also reported differences among physiological endpoints. Under increasing PBAT concentrations, lettuce MDA was highest at an intermediate dose, while proline and soluble protein increased mainly at the highest dose [33]. Higher biodegradable MP concentrations also produced stronger changes in rhizosphere microbial communities, soil metabolites, and microbial activity [34,35].

Particle size showed a less consistent relationship with plant responses. Most traits did not differ significantly among size classes, and smaller particles were not uniformly associated with stronger effects. In maize, smaller PU particles inhibited growth more strongly than larger particles, but the response to large particles also differed between soil types [16], indicating that particle-size effects can vary with soil conditions.

### 3.4. Effects of Environmental Factors on Plant Responses to MPs

Temperature showed the broadest influence among the environmental factors examined, with differences extending across growth, photosynthetic, and oxidative stress-related traits. Plant physiological processes are strongly temperature-dependent. Both low and high temperatures can impair photosynthetic function, while high temperature can affect PSII activity, carbon assimilation, ROS metabolism, and respiratory carbon loss [18,36,37,38]. Low temperature can also restrict chlorophyll synthesis and PSII repair [38]. Temperature can also influence the degradation of biodegradable plastics. PBAT and poly(lactic acid) have been shown to degrade faster at higher soil temperatures [39]. However, degradation rates were not reported consistently across the studies included in our meta-analysis and could not be evaluated separately from plant physiological responses. These differences may also reflect variation among studies in experimental setting, geographic location, season, and other conditions, so they cannot be attributed to temperature alone.

The subgroup and random forest analyses gave different results for SOM. Subgroup differences were limited mainly to a few growth traits, whereas the random forest ranked SOM highly for photosynthetic and oxidative stress-related responses. Organic matter can alter MP behavior through interactions with particle surfaces, aggregation, and soil structure, while also affecting nutrient availability and microbial activity [40,41]. Dissolved organic matter (DOM) can adsorb to MP surfaces and influence their mobility and bioavailability, and polymer–SOM interactions can further modify aggregation and transport in soils [40,41]. Rhizosphere processes may further modify these interactions. Wang et al. [42] found that MP-induced changes in DOM stability, microbial composition, and CO_2_ release were smaller in planted than in unplanted soil. The contrast between the subgroup and random forest results may partly reflect the coarse SOM classes used in the subgroup analysis and the influence of other soil and exposure variables.

Soil pH and the remaining exposure conditions showed weaker or more trait-specific effects. For pH, the clearest subgroup difference was observed for CAT, while most other traits changed little among pH classes. In a recent experiment, PE-MPs affected soil organic carbon fractions, microbial communities, and buckwheat growth differently in acidic, neutral, and alkaline soils, with plant growth responses also differing among soil types [43]. This suggests that pH effects may depend more on soil context than on a simple directional response along the pH gradient. Photoperiod and illumination affected only a few traits, and no significant differences were detected among exposure-duration groups.

### 3.5. Limitations and Future Directions

This study has several limitations. The available data allowed us to investigate the effects of several MP characteristics, but information on particle aging, shape, and surface properties was often limited. These characteristics can affect the behavior of MPs in soil and their effects on plant performance [44,45]. Replication was also uneven among some subgroup categories, and some subgroup estimates were based on only one independent study. Greater replication across comparable MP types, concentrations, particle sizes, and environmental conditions is needed to improve the reliability of subgroup comparisons. Soil-property information was incomplete for part of the database, particularly for nutrient variables, and studies reporting these properties may not fully represent the entire dataset. Many values were extracted from published figures using GetData Graph Digitizer, which may introduce minor measurement error.

Most available studies were conducted under controlled conditions, and the concentrations, exposure durations, and environmental settings used may not fully represent those in agricultural soils. Because soil and exposure conditions vary simultaneously in the field, responses observed under controlled conditions may not translate directly to agricultural soils. Longer-term experiments using field-relevant concentrations and better characterization of both soil conditions and MP properties would improve the environmental relevance of future studies.

The quantitative synthesis focused on the independent effects of MPs, whereas combined-exposure treatments were not pooled. This does not capture the more complex conditions in agricultural soils, where MPs frequently coexist with heavy metals, antibiotics, pesticides, and other contaminants. A separate synthesis of co-exposure studies would help distinguish additive, synergistic, and antagonistic responses across plant and soil endpoints. Future studies should also cover a wider range of crops and soil types and better link experimental exposure levels with MP occurrence in agricultural soils. These gaps currently limit the development of quantitative MP thresholds for agricultural soils.

## 4. Materials and Methods

### 4.1. Literature Search and Database Construction

We compiled a database of published studies to quantify plant responses to MP exposure and examine the key factors affecting these responses. Relevant studies were collected from the Web of Science Core Collection and China National Knowledge Infrastructure (CNKI) databases. The literature search included articles published up to September 2026. In the Web of Science database, the following search string was used: TS = (microplastic$ OR “plastic particle$” OR “plastic debris” OR polyethylene OR polypropylene OR polystyrene OR “polyvinyl chloride” OR polyamide OR polyurethane OR “polyethylene terephthalate” OR polycarbonate OR “polymethyl methacrylate” OR “ethylene vinyl acetate” OR “polylactic acid”) AND TS = (plant$ OR crop$) AND TS = (soil$ OR terrestrial$). Equivalent Chinese keywords were used for searches in CNKI.

Studies were included when they met the following criteria: (1) the experiment investigated the effects of MP exposure on terrestrial plants or crops; (2) both an MP treatment and a corresponding control without MP addition were available; (3) when a study included both MP-only and co-exposure treatments, only the MP-only treatment and its corresponding uncontaminated control were extracted; studies were excluded from the quantitative synthesis when the independent effect of MPs could not be separated from that of another contaminant; (4) at least one plant growth, photosynthetic, or oxidative stress-related trait was reported; (5) mean values, sample sizes, and standard deviations (SDs) or standard errors (SEs) were available; and (6) each treatment included at least three replicates. Studies involving aquatic plants, algae, field observations without controlled MP exposure, review articles, and studies lacking extractable quantitative data were excluded. After screening and data extraction, 100 independent studies (Table S1) comprising 2613 paired observations were retained for subsequent analyses.

For each eligible study, data were extracted from paired MP-treatment and control groups. When data were presented only in figures, values were digitized using GetData Graph Digitizer (version 2.22). Standard errors were converted to standard deviations before analysis.

Plant variables were grouped into three categories. Growth traits included plant height, root length, root biomass, and shoot biomass. Photosynthetic traits comprised chlorophyll *a*, chlorophyll *b*, net photosynthetic rate, stomatal conductance, transpiration rate, and intercellular CO_2_ concentration. Oxidative stress-related traits included the damage markers H_2_O_2_ and MDA, as well as antioxidant enzyme activities, including SOD, CAT, and POD.

For each observation, we also recorded plant group, MP characteristics (biodegradability, particle size, and concentration), soil properties (pH, SOM, nutrient status), and exposure conditions (temperature, illumination, photoperiod, and exposure duration). Soil nutrient status was represented by total and available NPK. Total NPK was calculated as the sum of TN, TP, and TK, whereas available NPK was calculated as the sum of available N, available P, and available K. These variables were included in subgroup analyses where data were available.

### 4.2. Meta-Analysis

The effect of MP exposure on each plant trait was expressed as the natural logarithm of the response ratio (ln*RR*):

(1) $$lnRR=ln\left(\frac{X_{t}}{X_{c}}\right)$$

where *X_t_* and *X_c_* represent the mean values of the MP treatment and control groups, respectively.

Because many studies contributed more than one effect size, multilevel meta-analytic models were fitted with study identity and effect size included as nested random effects, and model parameters were estimated using restricted maximum likelihood (REML) [46,47]. Pooled ln*RR* estimates were converted to percentage changes as follows:

(2) $$Percentage\;change\;\left(\%\right)=\left(e^{lnRR_{++}}-1\right)\times 100\%\;$$

Positive values indicate increases under MP exposure, whereas negative values indicate decreases. An effect was considered statistically significant when its 95% confidence interval did not include zero. Heterogeneity was summarized using variance components at the study and within-study levels and total *I*^2^ (Table S3).

Subgroup analyses were conducted within the same multilevel framework. Plant group, MP biodegradability, MP concentration, MP size, soil properties, and exposure conditions were included as moderators where data were available. Differences among subgroup levels were evaluated using moderator tests. The numbers of independent studies (*k*) and effect sizes (*n*) were recorded for each subgroup, and replication across subgroup levels is summarized in Table S4. Subgroup estimates based on only one independent study were interpreted with caution because they lacked between-study replication. A significant effect within one subgroup but not another was not considered evidence of a difference between the two subgroups unless the moderator test was significant (*p* < 0.05). As a sensitivity analysis, CR2-adjusted robust inference clustered by study was used to examine the influence of within-study dependence [48], and the results were compared with the multilevel estimates (Table S5). Soil-property subgroup analyses were conducted according to data availability; coverage for each trait group is reported in Table S6. Soil pH and SOM were not analyzed for photosynthetic traits because data coverage was insufficient. Total and available NPK were omitted because they were available from only five and six studies, respectively.

Small-study effects were assessed separately for growth, photosynthetic, and oxidative stress-related traits using Egger-type regression tests with study-clustered CR2 adjustment [49]. The Egger test indicated small-study effects for growth traits (*p* < 0.001), but not for photosynthetic (*p* = 0.095) or oxidative stress-related traits (*p* = 0.064). Rosenthal’s fail-safe numbers were 65,218, 17,119, and 60,728, respectively, all substantially exceeding the corresponding 5*k* + 10 criteria [50]. Thus, although small-study effects were detected for growth traits, the main conclusions were relatively robust to the potential influence of unpublished null results (Table S7). All meta-analyses were performed in R (version 4.5.2) using the metafor package, with CR2-adjusted inference obtained using the clubSandwich package.

### 4.3. Random Forest Modeling

Random forest regression models [51] were used to evaluate the relative importance of explanatory variables for variation in plant responses to MP exposure. Separate models were constructed for growth traits, photosynthetic traits, and oxidative stress-related traits. Individual effect sizes (ln*RR*) within each trait group were used as the response variable. Eight candidate predictors were included: MP size, MP concentration, SOM, soil pH, exposure duration, temperature, illumination, and photoperiod. The random forest models used the original continuous values of these variables. Total and available NPK were excluded because of their limited data coverage, which would have required extensive imputation. Because individual studies contributed multiple effect sizes, models were evaluated using repeated study-grouped five-fold cross-validation, repeated 10 times with Study_ID as the grouping variable. Missing values for the retained predictors were imputed within each training fold, and random forest hyperparameters, including mtry, were tuned by grouped cross-validation using the training data only.

Predictor importance was calculated on the held-out data using permutation importance based on the increase in MSE, with 20 permutations for each predictor. Negative importance values were set to zero before normalization, and positive values were normalized to sum to 100% within each fold. Figure 6 presents the mean normalized importance across valid outer folds. Predictor-ranking stability was evaluated from the untransformed permutation importance values using mean rank, rank interquartile range, and Top-3 and Top-5 frequencies. Model performance was described using cross-validated *R*^2^, RMSE, and MAE. Detailed model performance and ranking stability are provided in Tables S8 and S9.

## 5. Conclusions

Across the studies included in this meta-analysis, MPs affected the three trait groups to different extents. Oxidative stress markers and antioxidant enzyme activities showed the largest overall changes, followed by photosynthetic and growth traits. Differences between monocotyledons and dicotyledons were trait-specific: monocotyledons showed larger numerical reductions in some root traits and stronger POD and SOD responses, whereas dicotyledons showed a larger reduction in shoot fresh biomass. Among MP characteristics, higher concentrations were linked to stronger growth inhibition, while particle-size effects were less consistent and biodegradability showed few consistent differences across traits. For the environmental factors examined, temperature was associated with responses across all three trait groups and also ranked among the more important predictors in the random forest analysis. SOM and soil pH showed more limited or trait-specific effects, and exposure duration showed little overall influence. These results improve our understanding of MP effects on plants under different conditions and provide a basis for future assessments of MP effects in terrestrial plants.

## Figures and Tables

**Figure 1:**
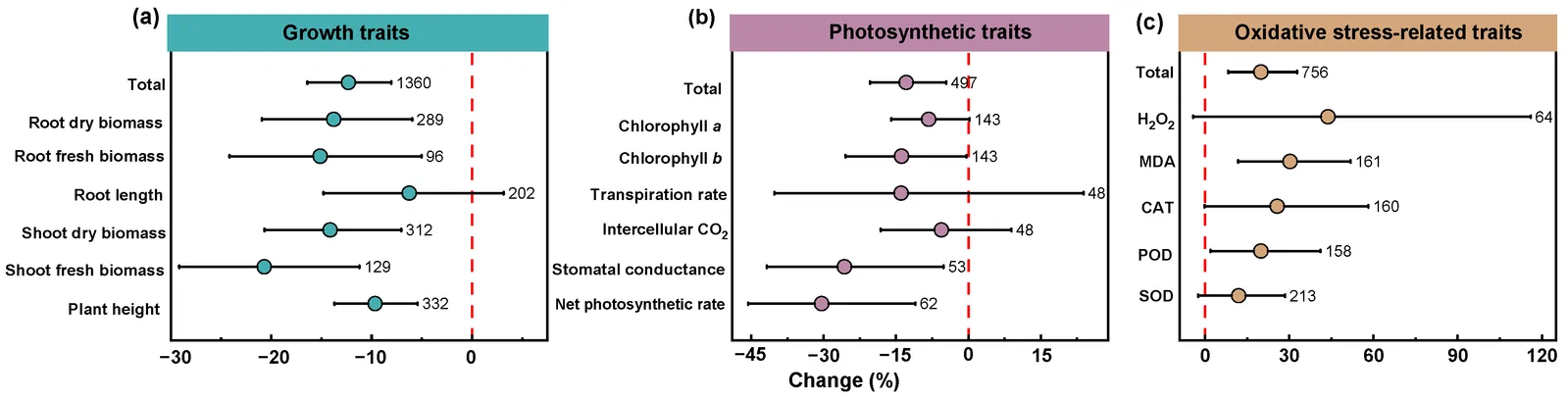
Effects of MPs on plant growth traits (**a**), photosynthetic traits (**b**), and oxidative stress-related traits (**c**). Points represent percentage changes relative to the control, and horizontal error bars indicate 95% confidence intervals. Red dashed line indicates no effect. Numbers beside the points indicate the number of effect sizes.

**Figure 2:**
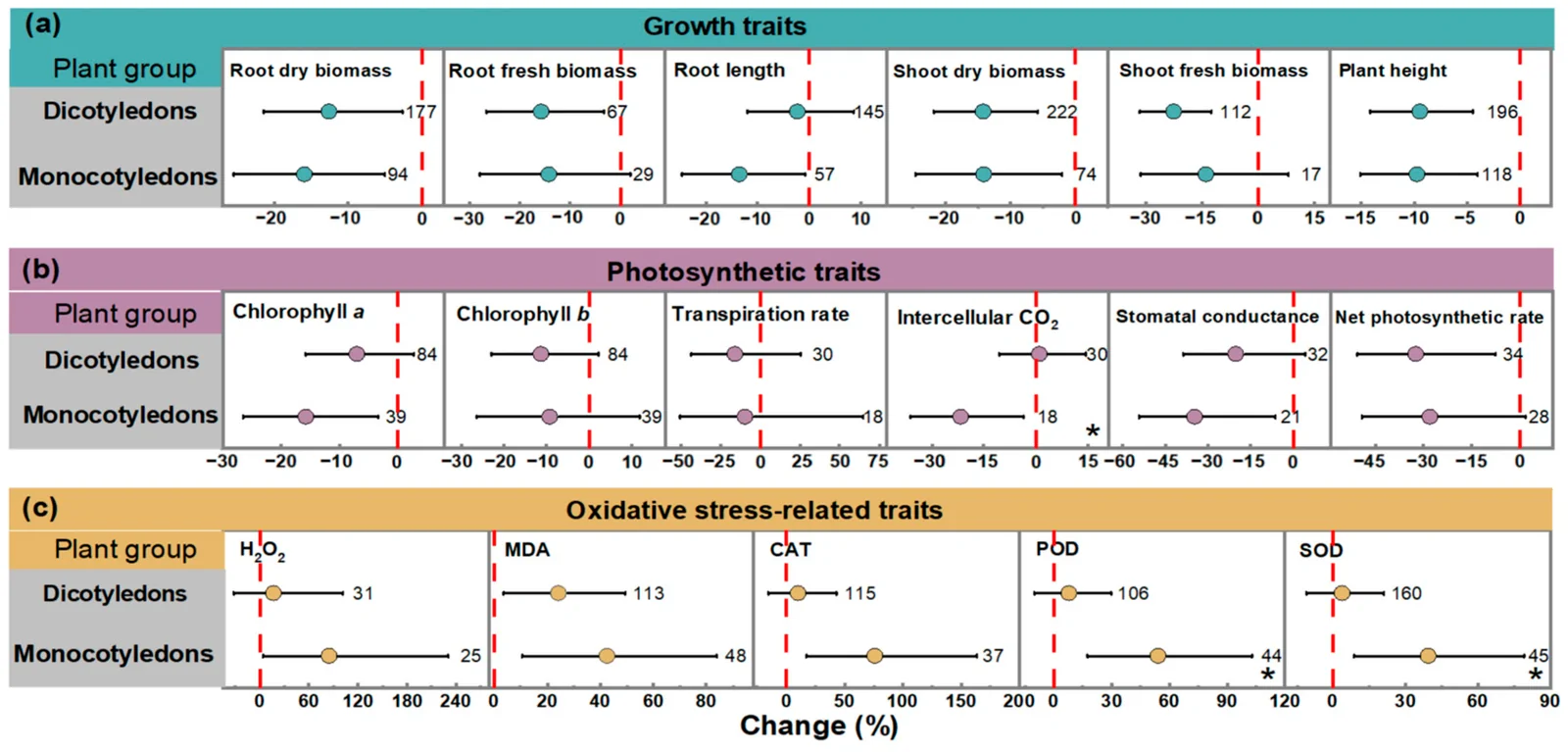
Effects of MPs on plant growth traits (**a**), photosynthetic traits (**b**), and oxidative stress-related traits (**c**) in dicotyledons and monocotyledons. Points represent percentage changes relative to the control, and horizontal error bars indicate 95% confidence intervals. Numbers beside the points indicate the number of effect sizes. Red dashed lines indicate no effect. Asterisks indicate significant differences between dicotyledons and monocotyledons based on omnibus *Q*_M_ tests (*p* < 0.05).

**Figure 3:**
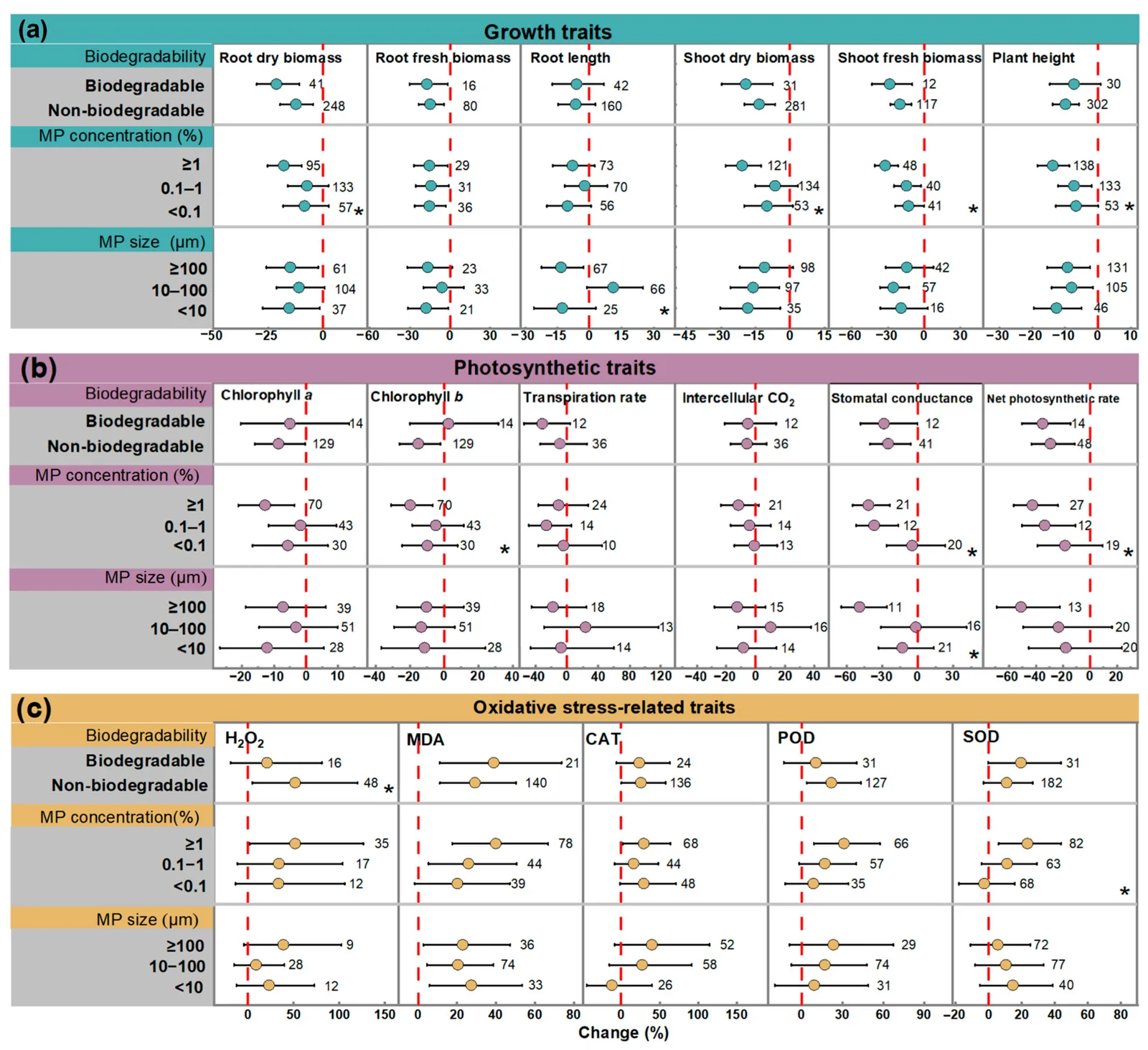
Effects of MP biodegradability, concentration, and size on growth traits (**a**), photosynthetic traits (**b**), and oxidative stress-related traits (**c**). Points represent percentage changes relative to the control, and horizontal error bars indicate 95% confidence intervals. Red dashed lines indicate no effect. Asterisks indicate significant differences among subgroups based on omnibus *Q*_M_ tests (*p* < 0.05). Numbers beside the points indicate the number of effect sizes.

**Figure 4:**
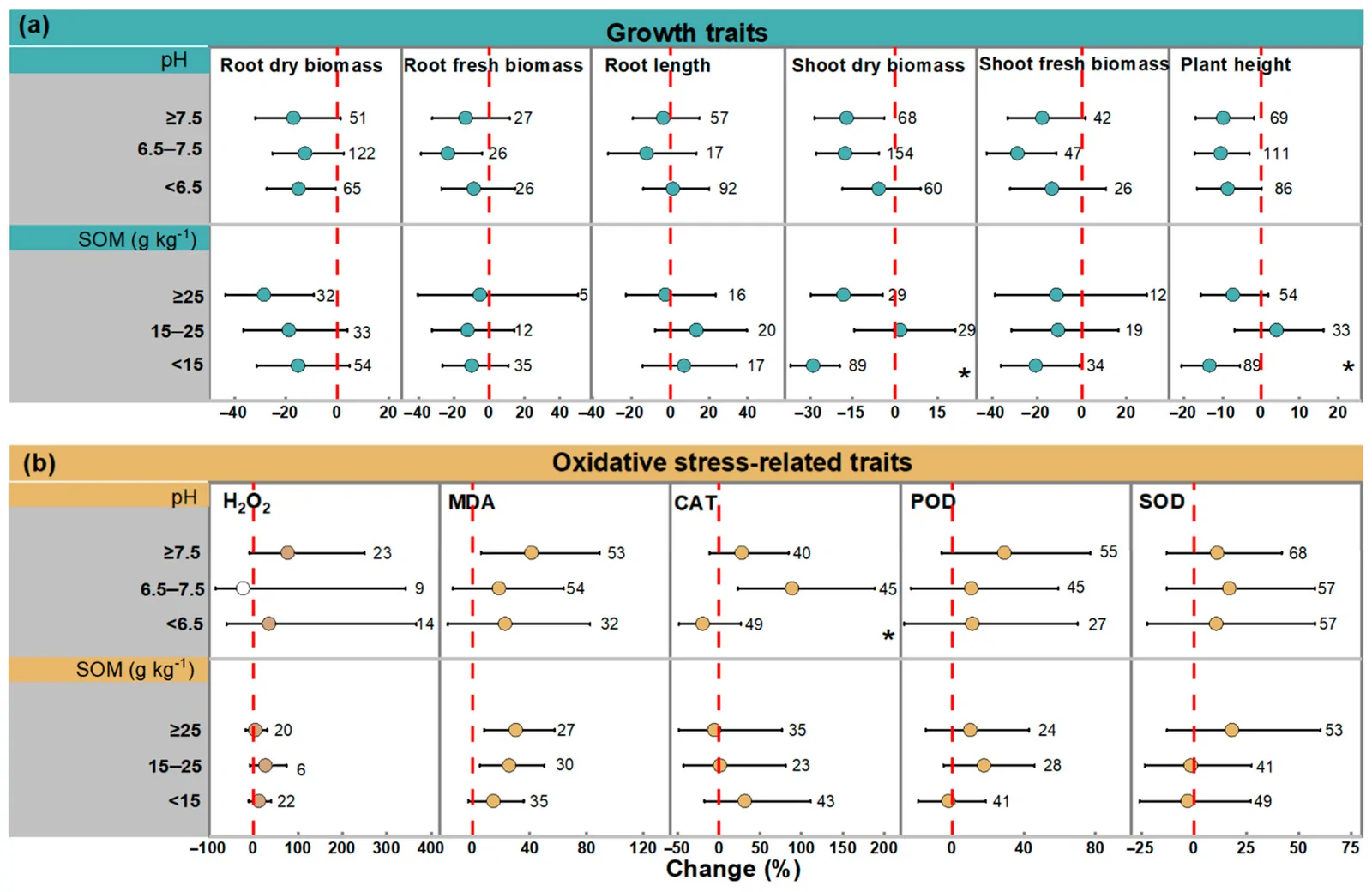
Effects of soil pH and SOM on plant growth traits (**a**) and oxidative stress-related traits (**b**) under MP exposure. Points represent percentage changes relative to the control, and horizontal error bars indicate 95% confidence intervals. Red dashed lines indicate no effect. Asterisks indicate significant differences among subgroups based on omnibus *Q*_M_ tests (*p* < 0.05). Hollow points indicate subgroup estimates based on only one independent study. Numbers beside the points indicate the number of effect sizes.

**Figure 5:**
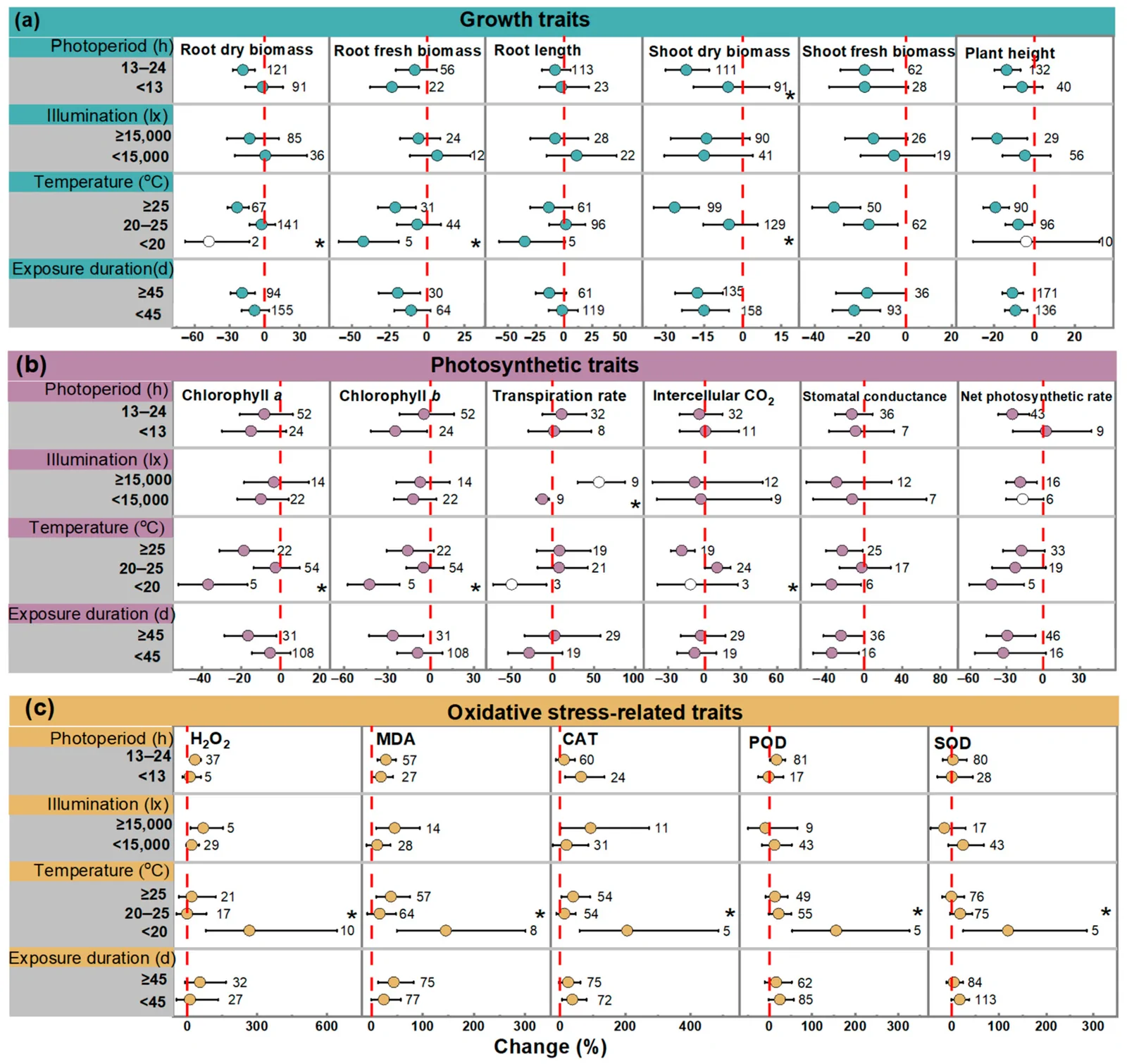
Effects of photoperiod, illumination, temperature, and exposure duration on plant growth traits (**a**), photosynthetic traits (**b**), and oxidative stress-related traits (**c**) under MP exposure. Points represent percentage changes relative to the control, and horizontal error bars indicate 95% confidence intervals. Red dashed lines indicate no effect. Asterisks indicate significant differences among subgroups based on omnibus *Q*_M_ tests (*p* < 0.05). Hollow points indicate subgroup estimates based on only one independent study. Numbers beside the points indicate the number of effect sizes.

**Figure 6:**
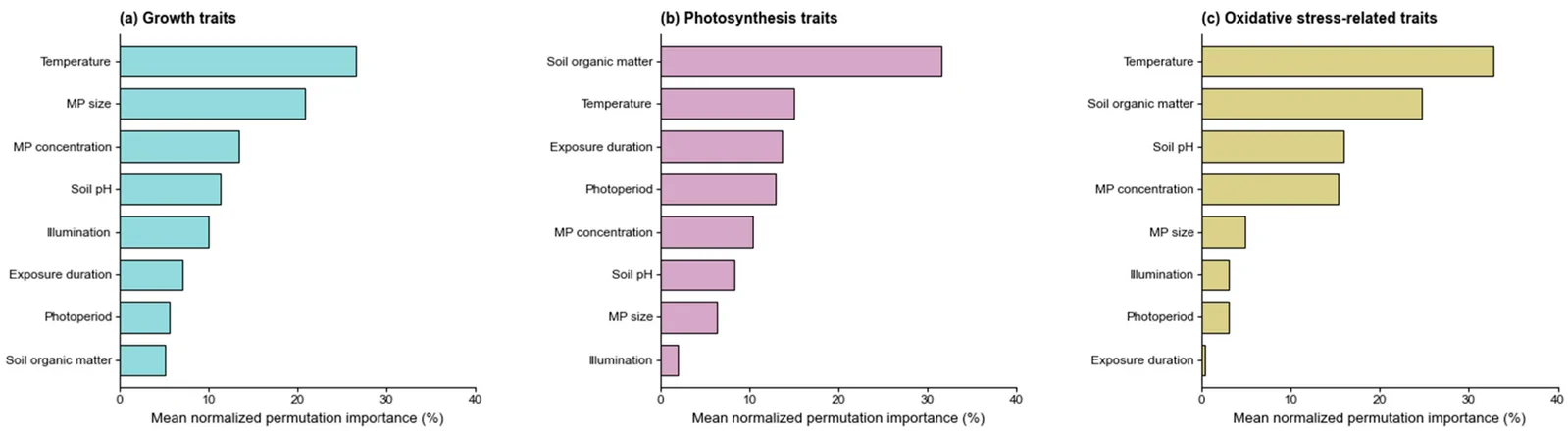
Relative importance of predictors of MP effects on growth traits (**a**), photosynthetic traits (**b**), and oxidative stress-related traits (**c**), based on random forest models. Predictor importance was evaluated on held-out data using permutation importance based on the increase in mean squared error. Negative values were set to zero, and the remaining importance values were normalized to sum to 100% within each fold.

## Data Availability

The original data supporting the conclusions of this article are included in the article and Supplementary Materials. The minimal dataset supporting the meta-analysis is provided as Data S1. Further inquiries can be directed to the corresponding author.

## Supplementary Materials

The following supporting information can be downloaded at: https://www.mdpi.com/article/10.3390/plants15192911/s1, Data S1: Minimal dataset used in the meta-analysis; Table S1: Studies included in the final meta-analysis; Table S2: Classification criteria used for subgroup analyses of plant responses to microplastic exposure; Table S3: Multilevel meta-analysis results and heterogeneity statistics; Table S4: Moderator tests and replication balance for subgroup analyses; Table S5: Sensitivity analysis comparing conventional multilevel and CR2-adjusted inference; Table S6: Availability of soil-property data by trait group; Table S7: Assessment of small-study effects by trait group; Table S8: Cross-validated performance of random forest models for the three plant trait groups; Table S9: Stability of predictor importance rankings across outer cross-validation folds.

## Abbreviations

The following abbreviations are used in this manuscript:

CAT	Catalase
CI	Confidence interval
H_2_O_2_	Hydrogen peroxide
MAE	Mean absolute error
MDA	Malondialdehyde
MP	Microplastic
MSE	Mean squared error
NPK	Nitrogen, phosphorus, and potassium
PBAT	Poly(butylene adipate-co-terephthalate)
PE	Polyethylene
POD	Peroxidase
PS	Polystyrene
PSII	Photosystem II
PU	Polyurethane
*R*^2^	Coefficient of determination
RMSE	Root mean squared error
ROS	Reactive oxygen species
SD	Standard deviation
SOD	Superoxide dismutase
SOM	Soil organic matter
TK	Total potassium
TN	Total nitrogen
TP	Total phosphorus
