# Supplementary material for: Effects of Microplastics on Growth, Photosynthesis, and Oxidative Stress-Related Traits in Terrestrial Plants: A Global Meta-Analysis
Source: Plants (Basel). 2026 Sep 23;15(19):2911. doi: 10.3390/plants15192911 (PMC13667275; doi:10.3390/plants15192911)
Supplement: Supplementary file 1 [file plants-15-02911-s001.zip › Supplementary Materials.pdf]

Supplementary Materials for

Effects of Microplastics on Growth, Photosynthesis, and Oxidative Stress-Related Traits in Terrestrial Plants: A Global Meta-Analysis

Jinling Xu [1, †, *], Dongwen Yu [1, †], Mufan Liu [2], Xuehan Zhang [1], Hangyu Liu [3] and Yuzhen Liu [1]

[1] College of Geography and Environment, Shandong Province Key Laboratory of Emerging Contaminants Risk Prevention and Control, Shandong Normal University, Jinan 250358, China; 18853596909@163.com (D.Y.); 2025215014@stu.sdnu.edu.cn (X.Z.); 110102@sdnu.edu.cn (Y.L.)

[2] College of Life Sciences, Shandong Normal University, Jinan 250358, China; 202513000237@stu.sdnu.edu.cn (M.L.)

[3] College of Natural Resources and Environment, Northwest A&F University, Yangling 712100, China; 06091836@nwafu.edu.cn (H.L.)

* Correspondence: xujinling@sdnu.edu.cn

[†] These authors contributed equally to this work.

Table S1. Studies included in the final meta-analysis.

No.	Study ID	Title	Year	DOI	Reference No.
1	S001	Combined exposure of PVC-microplastic and mercury chloride ($HgCl_2$) in sorghum (*Pennisetum glaucum* L.) when its seeds are primed titanium dioxide nanoparticles (TiO_2-NPs)	2024	10.1007/s11356-023-31733-9	[52]
2	S003	Co-exposure to tire wear particles and nickel inhibits mung bean yield by reducing nutrient uptake	2024	10.1039/D4EM00070F	[53]
3	S004	Exploring *Bacillus mycoides* PM35 efficacy in enhancing rice (*Oryza sativa* L.) response to different types of microplastics through gene regulation and cellular fractionation	2024	10.1007/s11356-024-33229-6	[54]
4	S005	Biodegradable microplastics affect the wheatgrass traits, Fe plaque development involved in Sb accumulation, and microbial community functions in antimony-contaminated riparian wetlands	2022	10.1021/acssuschemeng.1c08672	[55]
5	S006	Interactive effects of polystyrene microplastics and Pb on growth and phytochemicals in mung bean (*Vigna radiata* L.)	2023	10.1016/j.jhazmat.2023.130966	[56]
6	S007	Effects of microplastics and cadmium on the soil-wheat system as single and combined contaminants	2023	10.1016/j.plaphy.2023.01.023	[57]
7	S008	Field application of biodegradable microplastics has no significant effect on plant and soil health in the short term	2023	10.1016/j.envpol.2022.120556	[58]
8	S009	Can microplastics threaten plant productivity and fruit quality? Insights from Micro-tom and Micro-PET/PVC	2023	10.1016/j.scitotenv.2023.165119	[59]
9	S010	Comparative analysis of the effects of microplastics and nitrogen on maize and wheat: Growth, redox homeostasis, photosynthesis, and AsA-GSH cycle	2024	10.1016/j.scitotenv.2024.172555	[60]
10	S011	Increasing concentration of pure micro- and macro-LDPE and PP plastic negatively affect crop biomass, nutrient cycling, and microbial biomass	2023	10.1016/j.jhazmat.2023.131932	[61]
11	S012	Interactive impacts of microplastics and arsenic on agricultural soil and plant traits	2024	10.1016/j.scitotenv.2023.169058	[62]

12	S013	Combined effects of oxytetracycline and microplastic on wheat seedling growth and associated rhizosphere bacterial communities and soil metabolite profiles	2022	10.1016/j.envpol.2022.119046	[63]
13	S014	Modulations in protein phosphorylation explain the physiological responses of barley (*Hordeum vulgare*) to nanoplastics and ZnO nanoparticles	2023	10.1016/j.jhazmat.2022.130196	[64]
14	S015	Effect of different types and shapes of microplastics on the growth of lettuce	2023	10.1016/j.chemosphere.2023.139660	[65]
15	S016	Unveiling the detrimental effects of polylactic acid microplastics on rice seedlings and soil health	2024	10.1016/j.chemosphere.2024.141771	[66]
16	S017	Effect of co-presence of cadmium or procymidone with microplastic films in soil on lettuce growth	2023	10.1186/s13765-023-00827-w	[67]
17	S018	Impact of microplastics on bioaccumulation of heavy metals in rape (*Brassica napus* L.)	2022	10.1016/j.chemosphere.2021.132576	[68]
18	S019	Pesticide bioaccumulation in radish produced from soil contaminated with microplastics	2024	10.1016/j.scitotenv.2023.168395	[69]
19	S022	Translocation and chronic effects of microplastics on pea plants (*Pisum sativum*) in copper-contaminated soil	2022	10.1016/j.jhazmat.2022.129194	[70]
20	S023	Effects of polystyrene nanoplastics (PSNPs) on the physiology of *Allium sativum* L.: Photosynthetic pigments, antioxidant enzymes, phytohormones, and nutritional quality	2024	10.1016/j.envexpbot.2024.105654	[71]
21	S024	Single and composite damage mechanisms of soil polyethylene/polyvinyl chloride microplastics to the photosynthetic performance of soybean (*Glycine max* [L.] Merr.)	2023	10.3389/fpls.2022.1100291	[72]
22	S025	Effects of polyethylene and biodegradable microplastics on the physiology and metabolic profiles of dandelion	2024	10.1016/j.envpol.2024.124116	[73]
23	S026	Toxic effects and mechanisms of engineered nanoparticles and nanoplastics on lettuce (*Lactuca sativa* L.)	2024	10.1016/j.scitotenv.2023.168421	[74]
24	S027	Physiological responses of lettuce (*Lactuca sativa* L.) to microplastic pollution	2020	10.1007/s11356-020-09349-0	[75]

25	S028	Effects of polyethylene and polylactic acid microplastics on plant growth and bacterial community in the soil	2022	10.1016/j.jhazmat.2022.129057	[76]
26	S029	Soil microorganisms play an important role in the detrimental impact of biodegradable microplastics on plants	2024	10.1016/j.scitotenv.2024.172933	[77]
27	S030	The joint toxicity of polyethylene microplastic and phenanthrene to wheat seedlings	2021	10.1016/j.chemosphere.2021.130967	[78]
28	S034	Polyethylene microplastics alter soil microbial community assembly and ecosystem multifunctionality	2024	10.1016/j.envint.2023.108360	[79]
29	S035	Microplastic shape, polymer type, and concentration affect soil properties and plant biomass	2021	10.3389/fpls.2021.616645	[44]
30	S036	Response of common bean (*Phaseolus vulgaris* L.) growth to soil contaminated with microplastics	2021	10.1016/j.scitotenv.2020.142516	[80]
31	S037	Impact of polyvinyl chloride (PVC) microplastic on growth, photosynthesis and nutrient uptake of *Solanum lycopersicum* L. (Tomato)	2024	10.1016/j.envpol.2024.123994	[81]
32	S038	Microplastics in agricultural soil: Polystyrene fragments inhibit soil microbial and enzymatic activities but promote nutrient concentration of Cowpea (*Vigna unguiculata*)	2023	10.1016/j.hazadv.2023.100263	[82]
33	S039	Impact of polyethylene microplastics and copper nanoparticles: Responses of soil microbiological properties and strawberry growth	2023	10.1016/j.apsoil.2022.104773	[83]
34	S042	Polyethylene microplastic and nano ZnO co-exposure: Effects on peanut (*Arachis hypogaea* L.) growth and rhizosphere bacterial community	2024	10.1016/j.jclepro.2024.141368	[84]
35	S044	Effect of macro-and micro-plastics in soil on growth of Juvenile Lime Tree (*Citrus aurantium*)	2020	10.3934/environsci.2020033	[85]
36	S046	Response of peanut plant and soil N-fixing bacterial communities to conventional and biodegradable microplastics	2023	10.1016/j.jhazmat.2023.132142	[86]
37	S047	Different effects and mechanisms of polystyrene micro- and nano-plastics on the uptake of heavy metals (Cu, Zn, Pb and Cd) by lettuce (*Lactuca sativa* L.)	2023	10.1016/j.envpol.2022.120656	[87]

38	S049	Biochar relieves the toxic effects of microplastics on the root-rhizosphere soil system by altering root expression profiles and microbial diversity and functions	2024	10.1016/j.ecoenv.2024.115935	[88]
39	S050	Stimulation versus inhibition: The effect of microplastics on pak choi growth	2022	10.1016/j.apsoil.2022.104505	[89]
40	S051	Comparing the impact of microplastics derived from a biodegradable and a conventional plastic mulch on plant performance	2024	10.1016/j.scitotenv.2024.173265	[15]
41	S052	Integrating metabolomics and high-throughput sequencing to investigate the effects of tire wear particles on mung bean plants and soil microbial communities	2024	10.1016/j.envpol.2023.122872	[90]
42	S053	Effects of polyester microfibers (PMFs) and cadmium on lettuce (*Lactuca sativa*) and the rhizospheric microbial communities: A study involving physio-biochemical properties and metabolomic profiles	2022	10.1016/j.jhazmat.2021.127405	[91]
43	S054	Responses of maize (*Zea mays* L.) seedlings growth and physiological traits triggered by polyvinyl chloride microplastics is dominated by soil available nitrogen	2023	10.1016/j.ecoenv.2023.114618	[92]
44	S055	Wheat (*Triticum aestivum* L.) seedlings performance mainly affected by soil nitrate nitrogen under the stress of polyvinyl chloride microplastics	2024	10.1038/s41598-024-54838-8	[93]
45	S056	Middle concentration of microplastics decreasing soil moisture-temperature and the germination rate and early height of lettuce (*Lactuca sativa* var. *ramosa* Hort.) in Mollisols	2023	10.1016/j.scitotenv.2023.167184	[94]
46	S057	Dry–wet cycle changes the influence of microplastics (MPs) on the antioxidant activity of lettuce and the rhizospheric bacterial community	2023	10.1007/s11368-023-03479-x	[95]
47	S058	The degradability of microplastics may not necessarily equate to environmental friendliness: A case study of cucumber seedlings with disturbed photosynthesis	2024	10.3390/agriculture14010053	[96]
48	S059	Impact of microplastic particle size on physiological and biochemical properties and rhizosphere metabolism of *Zea mays* L.: Comparison in different soil types	2024	10.1016/j.scitotenv.2023.168219	[16]

49	S060	A new quantitative insight: Interaction of polyethylene microplastics with soil – microbiome - crop	2023	10.1016/j.jhazmat.2023.132302	[97]
50	S062	Polylactic acid microplastics have stronger positive effects on the qualitative traits of rice (*Oryza sativa* L.) than polyethylene microplastics: Evidence from a simulated field experiment	2024	10.1016/j.scitotenv.2024.170334	[98]
51	S063	Effects of microplastic type on growth and physiology of soil crops: Implications for farmland yield and food quality	2023	10.1016/j.envpol.2023.121512	[99]
52	S065	Impact of polyethylene and polylactic acid microplastics on growth, physio-biochemistry and metabolism in soybean (*Glycine max*)	2022	10.19674/j.cnki.issn1000-6923.20220209.007	[100]
53	S066	Effects of polyethylene microplastics exposure on growth and yield of *Capsicum annuum* L.	2023	10.11654/jaes.2023-0233	[101]
54	S067	Toxic effects of polyethylene microplastics on higher plant *Vicia faba*	2021	10.13232/j.cnki.jnju.2021.03.005	[102]
55	S068	Effects of combined stress of polyethylene and sulfamethazine on seed germination, seedling growth, and physiological characteristics of soybean	2023	10.13227/j.hjkx.202211033	[103]
56	S069	Effects of soil microplastics residue on soybean seedlings growth and the physiological and biochemical characteristics	2021	10.16036/j.issn.1000-2650.2021.01.007	[104]
57	S070	Effect of microplastics on the growth and physiological characteristics of alfalfa (*Medicago sativa*)	2023	10.11686/cyxb2022237	[105]
58	S072	Effect of polylactic acid microplastics on soil properties, soil microbials and plant growth	2023	10.1016/j.chemosphere.2023.138504	[106]
59	S073	Evidence for the transportation of aggregated microplastics in the symplast pathway of oilseed rape roots and their impact on plant growth	2024	10.1016/j.scitotenv.2023.169419	[107]
60	S074	Foliar-applied polystyrene nanoplastics (PSNPs) reduce the growth and nutritional quality of lettuce (*Lactuca sativa* L.)	2021	10.1016/j.envpol.2021.116978	[108]
61	S075	Impact of microplastics from polyethylene and biodegradable mulch films on rice (*Oryza sativa* L.)	2022	10.1016/j.scitotenv.2022.154579	[109]
62	S076	Impact of microplastics on growth, photosynthesis and essential elements in *Cucurbita pepo* L.	2022	10.1016/j.jhazmat.2021.127238	[110]

63	S077	Integrated effects of residual plastic films on soil-rhizosphere microbe-plant ecosystem	2023	10.1016/j.jhazmat.2022.130420	[111]
64	S078	Interactions of microplastics and cadmium on plant growth and arbuscular mycorrhizal fungal communities in an agricultural soil	2020	10.1016/j.chemosphere.2020.126791	[112]
65	S079	The toxicological effect on pak choi of co-exposure to degradable and non-degradable microplastics with oxytetracycline in the soil	2023	10.1016/j.ecoenv.2023.115707	[113]
66	S080	Earthworms modulate the toxicity effect of low-density polyethylene on plant development	2023	10.1007/s42729-023-01319-y	[114]
67	S081	Responses of rice (*Oryza sativa* L.) plant growth, grain yield and quality, and soil properties to the microplastic occurrence in paddy soil	2022	10.1007/s11368-022-03232-w	[115]
68	S083	Effects of combined microplastic and cadmium pollution on sorghum growth, Cd accumulation, and rhizosphere microbial functions	2024	10.1016/j.ecoenv.2024.116380	[116]
69	S084	Cascading effects from soil to maize functional traits explain maize response to microplastics disturbance in multi-nutrient soil environment	2024	10.1016/j.geoderma.2023.116759	[17]
70	S085	Dosage and exposure time effects of two micro(nono)plastics on arbuscular mycorrhizal fungal diversity in two farmland soils planted with pepper (*Capsicum annuum* L.)	2024	10.1016/j.scitotenv.2024.170216	[117]
71	S086	Effects of microplastics on photosynthesized C allocation in a rice-soil system and its utilization by soil microbial groups	2024	10.1016/j.jhazmat.2024.133540	[118]
72	S087	Reprogramming of microbial community in barley root endosphere and rhizosphere soil by polystyrene plastics with different particle sizes	2023	10.1016/j.scitotenv.2023.161420	[119]
73	S089	Effects of polystyrene, polyethylene, and polypropylene microplastics on the soil-rhizosphere-plant system: Phytotoxicity, enzyme activity, and microbial community	2024	10.1016/j.jhazmat.2023.133417	[120]
74	S090	Assessing microplastic-induced changes in sandy soil properties and crop growth	2023	10.3390/agriengineering5030096	[121]
75	S092	Short-term physiological and biometrical responses of *Lepidium sativum* seedlings exposed to PET-made microplastics and acid rain	2021	10.1016/j.ecoenv.2020.111718	[122]

76	S093	Low temperature tolerance is impaired by polystyrene nanoplastics accumulated in cells of barley (*Hordeum vulgare* L.) plants	2022	10.1016/j.jhazmat.2021.127826	[123]
77	S094	Microplastic residues in wetland ecosystems: Do they truly threaten the plant-microbe-soil system?	2021	10.1016/j.envint.2021.106708	[124]
78	S095	Effects of different commercial polyethylene microplastics with different particle sizes on seed germination and seedling growth of two vegetables	2022	10.7524/AJE.1673-5897.20211220001	[125]
79	S096	Effects of low-density polyethylene microplastics on soil nutrients, rice growth and physiological characteristics	2025	10.6041/j.issn.1000-1298.2025.06.060	[126]
80	S097	Soil phosphorus availability and potential mechanisms of microplastics-polluted soil by corn planting	2026	10.11689/sc.2025090401	[127]
81	S098	Effects of polyethylene microplastics with different particle sizes on soil nutrients, growth and quality of Chinese cabbage	2025	10.13872/j.1000-0275.2025.0162	[128]
82	S099	Physiological and ecological response characteristics and transcriptomic change characteristics of rice (*Oryza sativa*) under different microplastic stresses	2025	10.13227/j.hjkx.202401125	[129]
83	S100	Effect of organic fertilizers on the accumulation and distribution of polystyrene nanoplastics in cotton plants	2026	10.13227/j.hjkx.202501276	[130]
84	S101	Effects of microplastics on growth, chlorophyll content and photosystem II of wheat seedlings	2025	10.3969/j.issn.0488-5368.2025.03.007	[131]
85	S102	Effects of polyethylene microplastics with different particle sizes on photosynthesis, biomass and root characteristics of maize seedlings	2025	10.3724/aauj.2025124	[132]
86	S103	Biochar mitigates micro- and nano-plastics stress in *Arabidopsis thaliana* L. by modulating plant physiological responses and improving soil chemical and biochemical conditions	2026	10.1016/j.envexpbot.2026.106407	[133]
87	S104	Durum wheat under plastic pressure: genotype-specific adaptation to polystyrene nanoplastics	2026	10.1016/j.plaphy.2026.111497	[134]
88	S105	Nano- and microplastics increase the occurrence of bacterial wilt in tomato (*Solanum lycopersicum* L.)	2024	10.1021/acsnano.4c05875	[135]

89	S106	Negative effects of pristine and UV-aged nanoplastics on lettuce growth and soil microbes	2025	10.1016/j.apsoil.2025.106211	[136]
90	S108	Aging reduces the negative effect of low-density polyethylene microplastics on soil-plant-microbial systems	2026	10.1016/j.envpol.2025.127626	[137]
91	S109	Polystyrene nanoplastics distinctly impact cadmium uptake and toxicity in *Arabidopsis thaliana*	2024	10.1016/j.envpol.2024.124373	[138]
92	S110	Mechanistic insights into the effects of micro- and nano-plastics on cherry radish physiology and organic compound distribution at the soil–root interface	2026	10.1016/j.plaphy.2025.110933	[139]
93	S111	Sensitivity of sorghum (*Sorghum saccharatum*) and mustard (*Sinapis alba*) to soil levels of bio-based microplastics	2026	10.3390/su18062974	[140]
94	S112	Polyethylene microplastics exert dose-dependent effects on the growth, physiology, and rhizosphere microbiome of *Persicaria capitata*	2026	10.3390/biology15070573	[141]
95	S113	The absorption, immobilization, and response mechanism of *Leymus chinensis* to microplastics and nanoplastics	2025	10.1016/j.jhazmat.2025.140335	[142]
96	S114	When nano- and microplastics meet taro (*Colocasia esculenta*) roots: their size-dependent adsorption, penetration, and promotion on secondary wall reinforcement	2025	10.1021/acs.est.4c11230	[143]
97	S115	Effects of polystyrene nanoplastics on tomato plant growth, fruit yield and quality	2026	10.1016/j.foodchem.2026.148117	[144]
98	S116	Nitrogen supply neutralizes the nanoplastic-plant interaction in a coastal wetland	2024	10.1016/j.envres.2024.118572	[145]
99	S117	The combination of hyperspectral imaging, untargeted metabolomics and lipidomics highlights a coordinated stress-related biochemical reprogramming triggered by polyethylene nanoparticles in lettuce	2025	10.1016/j.scitotenv.2025.178604	[146]
100	S118	Microplastic uptake and impacts on crops under realistic exposure: implications for soil–plant systems	2026	10.1007/s11356-026-37686-z	[147]

Note: Study ID refers to the publication-level cluster identifier used in the final analytical dataset. The final quantitative synthesis included 100 independent studies. Studies containing co-contaminant treatments were retained only when an independent MP-only treatment and an uncontaminated control were available.

Table S2. Classification criteria used for subgroup analyses of plant responses to microplastic exposure.

Category	Variable	Subgroup classification
Plant group	Plant group	Monocotyledons; Dicotyledons
MP characteristics	Biodegradability	Biodegradable MPs; Non-biodegradable MPs
	Particle size (μm)	<10; 10–100; ≥100
	Concentration (%)	<0.1; 0.1–1; ≥1
Soil properties	Soil pH	<6.5; 6.5–7.5; ≥7.5
	Soil organic matter (SOM) (g kg^{-1})	<15; 15–25; ≥25
	Total NPK (g kg^{-1})	<1; 1–2.5; ≥2.5
	Available NPK (mg kg^{-1})	<20; 20–100; ≥100
Exposure conditions	Temperature (°C)	<20; 20–25; ≥25
	Illumination (lx)	<15,000; ≥15,000
	Photoperiod (h)	<13; 13–24
	Exposure duration (d)	<45; ≥45

Note: MP, microplastic; SOM, soil organic matter. Total NPK was calculated as TN + TP + TK, whereas available NPK was calculated as AN + AP + AK. The categories reproduce the predefined subgroup cut-offs used in the final analyses. No complete available-NPK observations fell in the 20–100 mg kg^{-1} class, so that level does not appear in the corresponding subgroup estimates. These cut-offs are analytical categories and should not be interpreted as biological thresholds.

Table S3. Multilevel meta-analysis results and heterogeneity statistics.

Trait group	Outcome	k	n	Effect (%)	CR2 p	τ^2_{study}	τ^2_{within}	I^2_{total} (%)
Overall	Growth	88	1360	−12.3	<0.001	0.041	0.038	95.770
	Oxidative stress-related	48	756	19.9	<0.001	0.105	0.144	98.810
	Photosynthesis	37	497	−12.9	0.004	0.063	0.061	98.540
Growth	Plant height	50	332	−9.7	<0.001	0.019	0.013	95.340
	Root dry biomass	47	289	−13.8	0.001	0.067	0.028	85.610
	Shoot dry biomass	46	312	−14.1	<0.001	0.049	0.060	95.970
	Root length	35	202	−6.3	0.179	0.060	0.027	95.660
	Shoot fresh biomass	24	129	−20.7	<0.001	0.053	0.030	90.570
	Root fresh biomass	23	96	−15.1	0.006	0.045	0.039	93.270
Oxidative stress-related	SOD	41	213	12.0	0.105	0.172	0.029	99.170
	MDA	35	161	30.3	0.001	0.172	0.055	97.860
	POD	34	158	19.9	0.030	0.183	0.064	97.090
	CAT	32	160	25.7	0.051	0.377	0.065	99.210
	H_2O_2	14	64	43.8	0.076	0.472	0.046	99.390
Photosynthesis	Chlorophyll *a*	26	143	−8.2	0.056	0.036	0.034	97.040
	Chlorophyll *b*	26	143	−13.9	0.045	0.111	0.041	97.550
	Net photosynthetic rate	14	62	−30.5	0.008	0.157	0.026	97.400
	Stomatal conductance	12	53	−25.7	0.022	0.115	0.051	95.790
	Intercellular CO_2	10	48	−5.6	0.385	0.034	0.009	98.330
	Transpiration rate	10	48	−14.0	0.375	0.228	0.069	98.960

Note: k, number of independent studies; n, number of effect sizes. Effect (%) = [exp(lnRR) − 1] × 100. The p values are based on CR2 cluster-robust inference with Study_ID as the clustering unit. τ^2_{study} represents the between-study variance component, τ^2_{within} represents the within-study variance component, and I^2_{total} (%) represents total heterogeneity.

Table S4. Moderator tests and replication balance for subgroup analyses.

Trait group	Trait	Moderator	Q_M	$p_{moderator}$	Min *k*	Max *k*
Growth	Root dry biomass	Plant group	0.277	0.599	20	27
	Root fresh biomass		0.025	0.875	9	14
	Root length		2.354	0.125	14	24
	Shoot dry biomass		0.000	0.986	15	31
	Shoot fresh biomass		0.640	0.424	5	19
	Plant height		0.008	0.931	21	31
Photosynthesis	Chlorophyll *a*		1.795	0.180	8	17
	Chlorophyll *b*		0.035	0.852	8	17
	Transpiration rate		0.036	0.849	3	7
	Intercellular CO_2		4.299	0.038	3	7
	Stomatal conductance		0.767	0.381	5	7
	Net photosynthetic rate		0.068	0.794	6	8
Oxidative stress-related	H_2O_2		1.276	0.259	6	7
	MDA		0.740	0.390	12	23
	CAT		3.653	0.056	9	22
	POD		4.520	0.034	10	23
	SOD		3.874	0.049	11	29
Growth	Root dry biomass	MP biodegradability	3.773	0.052	11	44
	Root fresh biomass		0.145	0.704	6	22
	Root length		0.006	0.939	9	33
	Shoot dry biomass		1.129	0.288	9	45
	Shoot fresh biomass		1.008	0.315	4	24
	Plant height		0.508	0.476	9	49
Photosynthesis	Chlorophyll *a*		0.194	0.660	3	25
	Chlorophyll *b*		2.806	0.094	3	25

Trait group	Trait	Moderator	Q_M	$p_{moderator}$	Min *k*	Max *k*
	Transpiration rate		2.553	0.110	3	9
	Intercellular CO_2		0.004	0.951	3	9
	Stomatal conductance		0.090	0.765	3	11
	Net photosynthetic rate		0.627	0.428	4	13
Oxidative stress-related	H_2O_2		4.057	0.044	5	13
	MDA		0.556	0.456	6	34
	CAT		0.033	0.856	8	31
	POD		0.791	0.374	9	32
	SOD		1.053	0.305	9	39
Growth	Root dry biomass		7.996	0.018	17	30
	Root fresh biomass		0.047	0.977	10	11
	Root length		4.322	0.115	15	25
	Shoot dry biomass		9.942	0.007	16	27
	Shoot fresh biomass		14.023	<0.001	12	13
	Plant height		9.351	0.009	14	34
Photosynthesis	Chlorophyll *a*		5.813	0.055	10	18
	Chlorophyll *b*		9.205	0.010	10	18
	Transpiration rate	MP concentration (%)	2.848	0.241	4	6
	Intercellular CO_2		2.662	0.264	5	6
	Stomatal conductance		11.733	0.003	5	7
	Net photosynthetic rate		7.512	0.023	5	7
Oxidative stress-related	H_2O_2		0.992	0.609	5	10
	MDA		3.074	0.215	15	21
	CAT		2.384	0.304	12	21
	POD		3.927	0.140	13	24
	SOD		10.301	0.006	16	26

Trait group	Trait	Moderator	Q_M	$p_{moderator}$	Min *k*	Max *k*
Growth	Root dry biomass	MP size (μm)	0.393	0.822	12	19
	Root fresh biomass		1.803	0.406	6	8
	Root length		19.209	<0.001	8	15
	Shoot dry biomass		0.792	0.673	12	17
	Shoot fresh biomass		1.111	0.574	4	12
	Plant height		1.137	0.566	13	20
Photosynthesis	Chlorophyll *a*		0.788	0.674	5	10
	Chlorophyll *b*		0.124	0.940	5	10
	Transpiration rate		2.436	0.296	2	5
	Intercellular CO_2		3.025	0.220	3	4
	Stomatal conductance		7.722	0.021	3	5
	Net photosynthetic rate		3.460	0.177	4	4
Oxidative stress-related	H_2O_2		1.307	0.520	3	6
	MDA		0.238	0.888	9	15
	CAT		3.421	0.181	7	12
	POD		0.390	0.823	7	14
	SOD		1.221	0.543	10	15
Growth	Root dry biomass	Soil pH	0.193	0.908	10	15
	Root fresh biomass		1.219	0.544	5	7
	Root length		0.879	0.644	5	12
	Shoot dry biomass		2.014	0.365	13	14
	Shoot fresh biomass		1.544	0.462	5	8
	Plant height		0.100	0.951	13	14
Photosynthesis	Chlorophyll *a*		1.271	0.530	5	10
	Chlorophyll *b*		0.060	0.970	5	10
	Transpiration rate		2.470	0.291	1	5

Trait group	Trait	Moderator	Q_M	$p_{moderator}$	Min *k*	Max *k*
	Intercellular CO_2		2.125	0.346	2	4
	Stomatal conductance		4.024	0.134	2	4
	Net photosynthetic rate		0.086	0.958	3	5
Oxidative stress-related	H_2O_2		0.827	0.661	1	7
	MDA		0.705	0.703	6	12
	CAT		7.212	0.027	7	11
	POD		0.496	0.780	6	12
	SOD		0.085	0.958	7	16
Growth	Root dry biomass		1.117	0.572	7	8
	Root fresh biomass		0.082	0.960	2	7
	Root length		0.885	0.642	5	6
	Shoot dry biomass		10.926	0.004	5	9
	Shoot fresh biomass		0.534	0.766	2	7
	Plant height		6.445	0.040	7	12
Photosynthesis	Chlorophyll *a*		1.093	0.579	2	9
	Chlorophyll *b*		2.829	0.243	2	9
	Transpiration rate	SOM (g kg^{-1})	0.776	0.378	1	3
	Intercellular CO_2		0.102	0.750	1	3
	Stomatal conductance		0.699	0.403	1	4
	Net photosynthetic rate		1.268	0.530	2	4
Oxidative stress-related	H_2O_2		0.982	0.612	2	5
	MDA		1.090	0.580	6	8
	CAT		0.845	0.655	5	9
	POD		1.637	0.441	6	9
	SOD		1.069	0.586	7	10
Growth	Root dry biomass	Total NPK (g kg^{-1})	1.199	0.273	1	2

Trait group	Trait	Moderator	Q_M	$p_{moderator}$	Min *k*	Max *k*
	Root fresh biomass		17.722	<0.001	1	4
	Root length		5.828	0.016	1	6
	Plant height		0.588	0.443	1	11
Oxidative stress-related	H_2O_2		113.381	<0.001	1	2
	MDA		171.855	<0.001	1	4
	CAT		5.573	0.018	1	4
	POD		29.101	<0.001	1	6
	SOD		33.671	<0.001	1	6
Growth	Root dry biomass		0.100	0.752	1	8
	Root length		1.958	0.162	1	6
Oxidative stress-related	H_2O_2		0.526	0.468	1	2
	MDA	Available NPK (mg kg^{-1})	0.018	0.894	1	5
	CAT		0.135	0.713	1	4
	POD		2.210	0.137	1	4
	SOD		5.062	0.024	1	4
Growth	Root dry biomass		3.300	0.069	9	19
	Root fresh biomass		1.961	0.161	6	10
	Root length		0.193	0.661	6	16
	Shoot dry biomass		3.855	0.0496	9	21
	Shoot fresh biomass		0.000	0.992	5	12
	Plant height	Photoperiod (h)	1.690	0.194	11	22
Photosynthesis	Chlorophyll *a*		0.392	0.531	5	9
	Chlorophyll *b*		2.014	0.156	5	9
	Transpiration rate		0.161	0.688	2	5
	Intercellular CO_2		0.127	0.722	3	5
	Stomatal conductance		0.040	0.841	2	6

Trait group	Trait	Moderator	Q_M	$p_{moderator}$	Min *k*	Max *k*
	Net photosynthetic rate		3.072	0.080	3	7
Oxidative stress-related	H_2O_2		0.730	0.393	2	7
	MDA		0.427	0.514	7	12
	CAT		2.860	0.091	6	12
	POD		1.076	0.300	5	16
	SOD		0.018	0.893	7	16
Growth	Root dry biomass		0.523	0.470	5	7
	Root fresh biomass		1.055	0.304	3	4
	Root length		0.906	0.341	6	6
	Shoot dry biomass		0.007	0.932	7	9
	Shoot fresh biomass		0.724	0.395	5	5
	Plant height		2.189	0.139	6	12
Photosynthesis	Chlorophyll *a*		0.359	0.549	3	4
	Chlorophyll *b*		0.168	0.682	3	4
	Transpiration rate	Illumination (lx)	31.118	<0.001	1	2
	Intercellular CO_2		0.026	0.871	2	2
	Stomatal conductance		0.229	0.632	2	2
	Net photosynthetic rate		0.048	0.826	1	3
Oxidative stress-related	H_2O_2		2.279	0.131	2	6
	MDA		2.156	0.142	3	7
	CAT		1.447	0.229	3	7
	POD		0.353	0.552	2	9
	SOD		2.118	0.146	4	9
Growth	Root dry biomass		11.583	0.003	1	16
	Root fresh biomass	Temperature (°C)	7.003	0.030	2	8
	Root length		4.143	0.126	2	14

Trait group	Trait	Moderator	Q_M	$p_{moderator}$	Min *k*	Max *k*
	Shoot dry biomass		9.259	0.002	15	17
	Shoot fresh biomass		3.537	0.060	9	12
	Plant height		5.899	0.052	1	17
Photosynthesis	Chlorophyll *a*		9.097	0.011	2	8
	Chlorophyll *b*		8.524	0.014	2	8
	Transpiration rate		5.505	0.064	1	4
	Intercellular CO_2		15.186	0.001	1	5
	Stomatal conductance		3.046	0.218	3	4
	Net photosynthetic rate		2.610	0.271	2	6
Oxidative stress-related	H_2O_2		8.410	0.015	3	4
	MDA		7.141	0.028	3	12
	CAT		7.766	0.021	2	11
	POD		8.201	0.017	2	10
	SOD		6.457	0.040	2	14
Growth	Root dry biomass		1.824	0.177	19	20
	Root fresh biomass		0.897	0.343	9	13
	Root length		1.375	0.241	13	17
	Shoot dry biomass		0.148	0.701	20	22
	Shoot fresh biomass		0.367	0.545	9	15
	Plant height		0.247	0.619	23	24
Photosynthesis	Chlorophyll *a*	Exposure duration (d)	1.676	0.195	8	17
	Chlorophyll *b*		1.862	0.172	8	17
	Transpiration rate		1.246	0.264	5	5
	Intercellular CO_2		0.156	0.693	5	5
	Stomatal conductance		0.336	0.562	4	7
	Net photosynthetic rate		0.039	0.843	4	10

Trait group	Trait	Moderator	Q_M	$p_{moderator}$	Min *k*	Max *k*
Oxidative stress-related	H_2O_2		0.500	0.480	4	8
	MDA		0.697	0.404	15	16
	CAT		1.135	0.287	13	16
	POD		0.145	0.703	14	17
	SOD		2.758	0.097	18	19

Note: Q_M and $p_{moderator}$ are the omnibus moderator-test statistics for differences among subgroup levels. Min *k* and Max *k* are the minimum and maximum numbers of independent studies contributing to the subgroup levels within each moderator analysis. Subgroup estimates based on only one independent study should be interpreted with caution because they lack between-study replication. A significant effect within one subgroup but not another was not considered evidence of a difference between the two subgroups unless the moderator test was significant ($p < 0.05$).

Table S5. Sensitivity analysis comparing conventional multilevel and CR2-adjusted inference.

Outcome type	Outcome	*k*	*n*	Multilevel *p*	CR2 *p*	CR2 df	Significance consistent
Trait group	Growth	88	1360	<0.001	<0.001	83.490	Yes
	Oxidative stress-related	48	756	<0.001	<0.001	46.020	Yes
	Photosynthesis	37	497	0.002	0.004	35.160	Yes
Growth	Plant height	50	332	<0.001	<0.001	45.930	Yes
	Root dry biomass	47	289	<0.001	0.001	43.930	Yes
	Shoot dry biomass	46	312	<0.001	<0.001	42.010	Yes
	Root length	35	202	0.170	0.179	32.440	Yes
	Shoot fresh biomass	24	129	<0.001	<0.001	21.170	Yes
	Root fresh biomass	23	96	0.002	0.006	20.800	Yes
Oxidative stress-related	SOD	41	213	0.097	0.105	39.390	Yes
	MDA	35	161	<0.001	0.001	33.490	Yes
	POD	34	158	0.023	0.030	32.060	Yes
	CAT	32	160	0.043	0.051	30.910	No
	H_2O_2	14	64	0.054	0.076	12.970	Yes
Photosynthesis	Chlorophyll *a*	26	143	0.045	0.056	24.280	No
	Chlorophyll *b*	26	143	0.034	0.045	24.500	Yes
	Net photosynthetic rate	14	62	0.001	0.008	12.190	Yes
	Stomatal conductance	12	53	0.007	0.022	10.560	Yes
	Intercellular CO_2	10	48	0.361	0.385	8.830	Yes
	Transpiration rate	10	48	0.350	0.375	8.930	Yes

Note: *k*, number of independent studies; *n*, number of effect sizes. The conventional multilevel and CR2 analyses used the same point estimates. CR2-adjusted inference used study-clustered robust standard errors with Satterthwaite-adjusted degrees of freedom. “Significance consistent” indicates whether the statistical conclusion at $p < 0.05$ was unchanged after CR2 adjustment.

Table S6. Availability of soil-property data by trait group.

Variable	Trait group	Studies available/total	Study coverage (%)	Effects available/total	Effect coverage (%)
Available NPK	Growth	16/88	18.2	172/1360	12.6
	Photosynthesis	6/37	16.2	76/497	15.3
	Oxidative stress-related	6/48	12.5	144/756	19.0
SOM	Growth	47/88	53.4	612/1360	45.0
	Photosynthesis	20/37	54.1	190/497	38.2
	Oxidative stress-related	29/48	60.4	477/756	63.1
Total NPK	Growth	13/88	14.8	118/1360	8.7
	Photosynthesis	5/37	13.5	63/497	12.7
	Oxidative stress-related	7/48	14.6	88/756	11.6
pH	Growth	74/88	84.1	1146/1360	84.3
	Photosynthesis	32/37	86.5	438/497	88.1
	Oxidative stress-related	37/48	77.1	628/756	83.1

Note: Study availability is the number of independent studies with non-missing data divided by the total number of studies in the corresponding trait group; effect-size availability is defined analogously. Coverage values describe data availability only and are not weights or measures of evidence quality.

Table S7. Assessment of small-study effects by trait group.

Trait group	k	n	Egger slope	CR2 p	Rosenthal fail-safe N	$5k + 10$ criterion
Growth	88	1360	-0.501	<0.001	65,218	450
Photosynthesis	37	497	-0.557	0.095	17,119	195
Oxidative stress-related	48	756	0.430	0.064	60,728	250

Note: k, number of independent studies; n, number of effect sizes. Egger-type tests were conducted using CR2-adjusted inference clustered by study. A significant CR2 p value ($p < 0.05$) was interpreted as evidence of small-study effects rather than definitive evidence of publication bias. Rosenthal's fail-safe N was compared with the conventional $5k + 10$ criterion.

Table S8. Cross-validated performance of random forest models for the three plant trait groups.

Trait group	*n*	*k*	Cross-validation design	R^2	RMSE	MAE
Growth	1360	88	10 repeats × 5 grouped folds	−0.043	0.369	0.255
Photosynthesis	497	37	10 repeats × 5 grouped folds	−0.234	0.425	0.319
Oxidative stress-related	756	48	10 repeats × 5 grouped folds	−0.173	0.575	0.382

Note: Models were evaluated by repeated study-grouped five-fold cross-validation. All effect sizes from the same study were assigned to the same fold. R^2, RMSE, and MAE were calculated on held-out observations.

Table S9. Stability of predictor importance rankings across outer cross-validation folds.

Trait group	Predictor	Mean rank	IQR	Top-3 frequency (%)	Top-5 frequency (%)
Growth	Temperature	3.00	3.75	68	80
	MP concentration	3.52	2.75	64	78
	MP size	3.76	5.00	50	68
	Soil pH	4.38	3.00	44	60
	Illumination	5.06	3.75	26	52
	Exposure duration	5.36	3.00	20	54
	Photoperiod	5.44	3.00	12	52
	SOM	5.48	2.75	16	56
Photosynthesis	SOM	2.80	2.00	76	82
	MP concentration	4.22	2.00	36	76
	Exposure duration	4.38	3.00	34	68
	Temperature	4.44	4.75	42	56
	Photoperiod	4.48	3.00	38	64
	Illumination	4.76	2.00	24	66
	Soil pH	5.40	5.00	28	46
	MP size	5.52	3.00	22	42
Oxidative stress-related	Temperature	2.68	3.00	72	86
	SOM	3.18	3.00	68	78
	Soil pH	3.76	3.00	54	80
	MP concentration	3.78	3.00	54	80
	Photoperiod	4.84	2.00	16	72
	Illumination	5.22	2.00	14	48
	MP size	5.48	3.00	20	44
	Exposure duration	7.06	1.00	2	12

Note: Predictor ranks were calculated across the completed outer cross-validation folds using permutation importance. Lower mean ranks indicate greater relative importance. IQR represents the interquartile range of predictor ranks across folds and reflects ranking variability. Top-3 and Top-5 frequencies indicate the percentages of outer folds in which a predictor ranked among the three or five most important predictors, respectively. Ranking statistics were calculated from the untransformed permutation importance values. For Figure 6, negative importance values were truncated to zero before normalization.
